# Protoplast Dissociation and Transcriptome Analysis Provides Insights to Salt Stress Response in Cotton

**DOI:** 10.3390/ijms23052845

**Published:** 2022-03-05

**Authors:** Qiankun Liu, Pengtao Li, Shuang Cheng, Zilin Zhao, Yuling Liu, Yangyang Wei, Quanwei Lu, Jiangping Han, Xiaoyan Cai, Zhongli Zhou, Muhammad Jawad Umer, Renhai Peng, Baohong Zhang, Fang Liu

**Affiliations:** 1 Zhengzhou Research Base, State Key Laboratory of Cotton Biology, School of Agricultural Sciences, Zhengzhou University, Zhengzhou 450001, China; liuthundering@163.com (Q.L.); hanjiangping2021@163.com (J.H.); 2 College of Biology and Food Engineering, Anyang Institute of Technology, Anyang 455000, China; lipengtao1056@126.com (P.L.); chengshuang0427@163.com (S.C.); zzl2435584587@163.com (Z.Z.); liuylay2012@163.com (Y.L.); weiyangyang511@126.com (Y.W.); daweianyang@163.com (Q.L.); aydxprh@163.com (R.P.); 3 State Key Laboratory of Cotton Research, Chinese Academy of Agricultural Sciences, Anyang 455000, China; cxycri@163.com (X.C.); zhonglizhou@163.com (Z.Z.); 2017Y90100110@caas.cn (M.J.U.); 4 School of Life Sciences, Zhengzhou University, Zhengzhou 450001, China; 5 Department of Biology, East Carolina University, Greenville, NC 27858, USA

**Keywords:** cotton, salt stress, RNA-seq, protoplast dissociation

## Abstract

As one of the pioneer crops widely planted in saline-alkaline areas, *Gossypium* provides daily necessities, including natural fiber, vegetable proteins, and edible oils. However, cotton fiber yield and quality are highly influenced by salt stress. Therefore, elucidating the molecular mechanisms of cotton in response to salinity stress is importance to breed new cultivars with high tolerance. In this study, we first developed a method for single-cell RNA-seq based on isolating protoplast from cotton root tips; then, we studied the impact of salinity stress on gene expression profiling and their dynamic changes using the developed high-efficiency method for protoplast dissociation suitable for single-cell RNA-seq. A total of 3391 and 2826 differentially expressed genes (DEGs) were identified in salt-treated samples before and after protoplast dissociation, respectively, which were enriched into several molecular components, including response to stimulus, response to stress, and cellular macromolecule metabolic process by gene ontology (GO) analysis. Plant hormone signal transduction, phenylpropanoid biosynthesis, and MAPK signaling pathway were found to be enriched via Kyoto Encyclopedia of Genes and Genomes (KEGG) analysis. Twenty-two and nine salinity-responsive DEGs participated in plant hormone signaling and MAPK signaling in roots, before and after protoplast dissociation, respectively; six upregulated DEGs were involved in ABA signaling transduction, namely, *Ga04G2111*, *Ga07G0142*, *Ga09G2061*, *Ga10G0262*, *Ga01G0063*, and *Ga08G1915* which indicates their potential functions on plants adapting to salt stress. Additionally, 384 and 257 transcription factors (TFs) were differentially expressed in salt-stress roots before and after protoplast dissociation, respectively, of which significantly up-regulated TFs mainly belonged to the AP2/ERF-ERF family, which implied their potential roles responding to salt stress. These results not only provide novel insights to reveal the regulatory networks in plant’s root response to salt stress, but also lay the solid foundation for further exploration on cellular heterogeneity by single-cell transcriptome sequencing.

## 1. Introduction

During each stage of the life cycle, the growth and development of plants are uninterruptedly affected by various biotic and abiotic stresses. Among abiotic stresses, drought and salt stresses are the leading contributors for yield loss. As increasing water scarcity and soil salinization, drought and salinity stress caused about 45% of the global loss of crop production [1]. Plenty of attempts had been conducted to investigate the biological processes during plant response to salt stress; results indicated that, in the initial stages, plants underwent water loss, which inhibited leaf growth; subsequently, osmotic effect could be quickly observed, which gradually inhibited cell division and cell growth, and finally led to stomatal closure [2]. Under salt stress, plants undergo osmotic stress and ion stress, of which the former inhibits the growth of young leaves, while the latter promotes the senescence of mature leaves. During the long history of evolution, plants have developed three strategies to cope the serious threats by salt stress, namely osmotic stress tolerance, Na^+^ Cl^−^ exclusion, and Na^+^ Cl^−^ accumulation. Additionally, salt stress could promote the transport of abscisic acid (ABA) to guard cells, resulting in reduced stomatal conductance and, thus, further reducing photosynthesis and photoinhibition to resist stress, which inhibits plants growth and leads to leaf senescence. Salt stress also induced oxidative stress by triggering high accumulation of reactive oxygen species in the aerobic process of cells, which plays an important role in maintaining homeostasis for protecting plants against abiotic stress, including salinity stress [3].

As one of the most important economic crops in the world, cotton not only provides the main natural fibers as the raw materials for textile mills, but also produces vegetable proteins and edible oils, which makes a significant contribution for world economic development. *Gossypium arboreum* (A_2_), a diploid ancestor of cultivated cotton species, was probably domesticated in Madagascar or in the Indus Valley (Mohenjo Daro) and subsequently dispersed to other parts of Africa and Asia [4], which was introduced into China more than 1000 years ago as an ornamental plant. During the long-term process of ecological adaptation and human selection, *G. arboreum* had developed into a unique geographical race called ‘Sinense cotton’ in China. Despite *G. arboreum* harbors many merits of precocity, strong resistance, and high strength, of which either fiber yield (boll weight and lint percentage) or quality (length, strength, and fineness) is seriously affected by salt stress [5]. In order to maintain the normal growth against salt stress, cotton has evolved several signaling pathways to respond to water deficit conditions [6], such as ABA, which is involved in the regulation of plant development, reproduction, and response to stresses. Dynamic changes in transpiration and stress pressure by water deficit or salt stress, osmotic stress refers to the effective reduction of water content [7], initiating that this signaling pathway could promote ABA synthesis, thus, inducing downstream gene expression and triggering various physiological and biochemical changes [8]. ABA signal transduction pathway plays a core role in plants responding to drought stress and salt stress [9,10,11]. Therefore, it is important to investigate the ABA signaling in cotton under salt stress and to screen the specific salt-tolerance genes, which will lay a solid foundation for breeding novel salt-resistant varieties harboring high yield and superior fiber quality. 

Transcription factors (TFs) such as AP2/EREBP, NAC, WRKY, MYB, bZIP, GRAS, and ERF [12,13] are key switching proteins capable of regulating the expression of downstream genes [12,14], which have been found to participate in inhibiting salt stress in plants, so as to adapt plants to stress-treated environments [15]. Hence, studying the changes in the expression patterns of TFs is of great significance for revealing the regulatory networks of plants in response to salt stress. 

Cell walls constitute the first barrier that protects plant cells, which not only makes them different from animal cells, but also increases difficulties in performing physiological, biochemical, and molecular-biology experiments. After removing cell walls, protoplasts could be obtained from plant cells, and their integrity and cell totipotency determine to be an ideal single-cell system. Therefore, protoplasts have been widely applied in the transient genetic transformation and single-cell RNA-seq (sc-RNA-seq) research, of which the former is devoted to conduct subcellular localization and interaction protein identification on targeted genes, and the later focus on revealing the cellular heterogeneity at the single cell level. Currently, protoplast dissociation has been successfully obtained from different tissues of *Arabidopsis* [16,17,18,19,20,21,22,23], maize [24], peanut [25], rice [26] and poplar [27], most of which were subsequently utilized to perform scRNA-seq for cell type identification. To date, numerous protoplast-based studies in cotton have been done on leaves, while few studies focused on the optimization method of protoplast isolation and its transcriptome analysis under salt stress at the single cell level in roots. 

In this study, we first explored the best method to dissociate the root protoplasts from *G. arboreum* under the normal growth conditions and salt stress, and then obtained high-quality protoplasts for single-cell transcriptome sequencing, which lays a solid foundation for our further cellular heterogeneity research in virtue of scRNA-seq. Bulk RNA-seq was chosen to conduct comparative analyses on gene expression of cotton roots before and after protoplast dissociation, and differentially expressed genes (DEGs) were subsequently subjected to enrichment annotation by GO and KEGG analyses. Together with transcription factor identification, some signaling pathways and salt-tolerance genes were identified in response to salt stress in this study. Current results expand our horizons on investigation of molecular mechanisms of salt resistance in cotton. Herein, the reported key gene candidates can be of immense importance for breeding high-yield, superior-fiber, salt-resistant cotton varieties in the future. 

## 2. Results

### 2.1. Highly-Efficient Method for Isolating Protoplasts from Cotton Roots 

**Figure 1:**
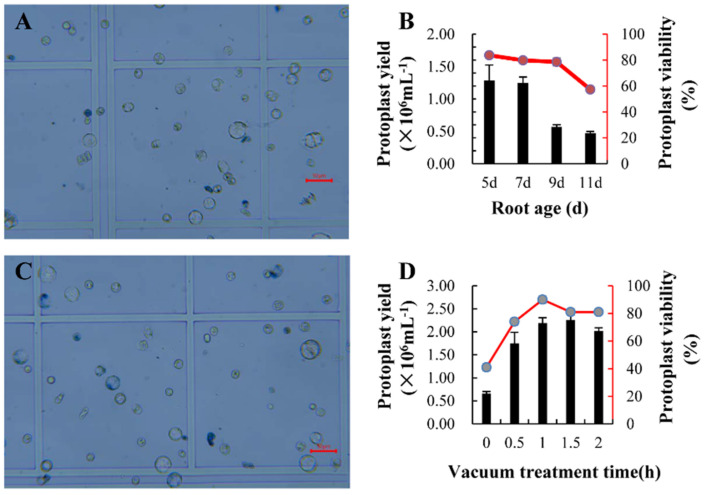
Effects of age and vacuum treatment time in protoplast isolation from *G. arboreum* lateral root tips. (**A**) The protoplasts stained with Trypan blue solution, which were isolated from 5-day-old lateral root tips. Scale bar, 50 μm; (**B**) Effect of root age on the yield and viability of protoplasts isolated from the cotton lateral root tips; data presented as means of three biological replicates with error bars indicating standard deviations (SD). The bar graph represents protoplast yield (×10^6^) and the red broken line graph represents protoplast viability (%); (**C**) The protoplasts stained with Trypan blue solution, which were isolated from the lateral root tips, were treated by vacuum for 1 h. Scale bar, 50 μm; (**D**) Effect of vacuum treatment time on the yield and viability of protoplasts isolated from the cotton lateral root tips; data presented as means of three biological replicates with error bars indicating standard deviations (SD). The bar graph represents protoplast yield (×10^6^) and the red broken line graph represents protoplast viability (%). 4.6 cm left.

Tissue age may affect the quantity and quality of isolated protoplasts. To harvest the best quality of protoplast with high yield, we compared the yield and cell viability of protoplasts isolating from 5-, 7-, 9- and 10-day-old root tips. Our results showed that 5-day-old root tips gave the high yield of protoplasts with highest viability (Figure 1A,B). Five-day-old root-generated protoplasts acquired more than 85% of cell viability which met the optimum standard for further scRNA-seq (Figure 1A,B). Thus, 5-day-old root tips were proved in this study to be the most proper growth stage for protoplasts isolation and transcriptome sequencing. 

In order to reduce the dissociation time and obtain all types of root cells, 0.05 MPa vacuum was set to make the enzymolysis solution blend in the sample interior, which could ensure that the obtained protoplasts have both high viability and integrity. The different time of vacuum treatments was designed to confirm the best conditions for protoplast dissociation, and root tips under 1 h vacuum treatment generated the most protoplast yield and the highest viability (Figure 1C,D). Although high protoplast yields were also observed under 1.5 h vacuum treatment, 85% of cell viability was only obtained under 1 h vacuum treatment. Therefore, 1 h vacuum treatment was chosen in isolating protoplasts from cotton root tips and later for transcriptome sequencing.

The effects of enzyme digestion time (2–8 h) were also investigated on protoplast isolation to evaluate their efficiency and protoplasts viability. Protoplast yield gradually increased as digestion time propelled from 2 h to 6 h, with a peak of 2.00 × 10^6^ protoplasts g^−1^ fresh weight at 6 h, and then decreased along with digestion time (Supplementary Figure S1A–D). Additionally, the cell viability of protoplasts obtained after 8 h enzymatic hydrolysis could not meet transcriptome sequencing standards. For obtaining highest cell viability, 6 h of enzymatic hydrolysis was approved to be the most proper digestion time for protoplast isolation and transcriptome sequencing.

**Figure 2:**
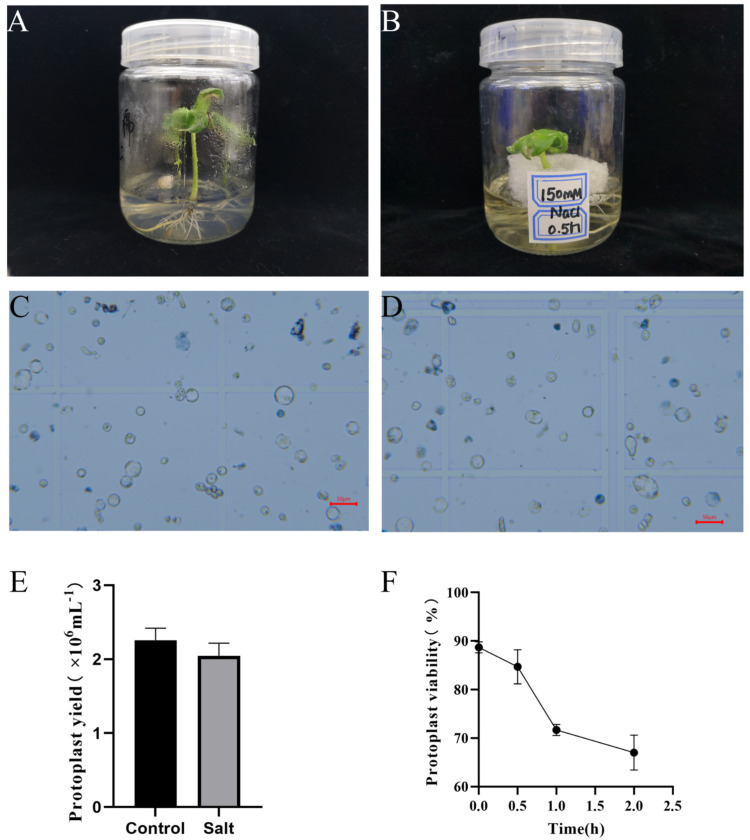
Effects of 150 mM NaCl treatment time on protoplast isolation from *G. arboreum* lateral root tips. The visual appearance of (**A**) control and (**B**) salt-stressed cotton seedlings after treatment of 150 mM NaCl for 0.5 h; The protoplasts stained with Trypan blue solution isolation from (**C**) control and (**D**) salt-stressed cotton seedlings. Scale bar, 50 μm; (**E**) The protoplast yield of control and salt-stressed cotton seedlings lateral root tips; (**F**) The protoplast viability under 150 mM NaCl treatment at different treatment times.

Based on the previous studies [28,29], 150 mM NaCl was regarded as a better concentration to study cotton plant response to salinity stress (Figure 2A). Here, we detected the effect of 150 mM NaCl on protoplast isolation from cotton root tips at different time intervals (0.5 h, 1 h and 2 h of treatments). After enzyme dissociation and cell viability tests (Figure 2B), the results showed that salt stress could affect the protoplast yield by ten thousand times; fortunately, the samples met the requirements for transcriptome sequencing (Figure 2C). By comparing the dissociated results at different time intervals, 150 mM NaCl treatment for 0.5 h on cotton seedlings obtained more than 85% of cell viability, while their viabilities were yet 71% and 67% under 150 mM NaCl treatment for 1 h and 2 h, respectively, of which the latter two could apparently not fit in the standard requirements for transcriptome sequencing (Figure 2D). In consequence, non-salt treatment and 150 mM NaCl treatment for 0.5 h were chosen as control and salt-stress groups, and fresh root tips and dissociated protoplasts from root tissues were also sampled to perform RNA-seq, respectively. After statistics analyses, the numbers of protoplasts in the control group for transcriptome sequencing was 2.26 × 10^6^/mL with 89% cell viability, whereas it was 2.05 × 10^6^/mL with 85% cell viability in the salt treatment group (Figure 2C).

### 2.2. Identification and Enrichment Analyses of DEGs

A total of 12 cDNA libraries with 3 biological replicates were constructed for RNA-seq to explore the molecular mechanism of cotton root response to salt stress and the differences before and after protoplasmic preparation. Each library generated 46.63–51.72 million raw reads with an average of 49.09 ± 1.66 million raw read per library (Table 1). After sequence cleanup, 573.24 million clean reads were obtained, of which the Q30 was more than 91.85% and the overall GC contents were about 44.36%. The majority of sequences were mapped to the reference genome with about 90% matched ratio.

**Figure 3:**
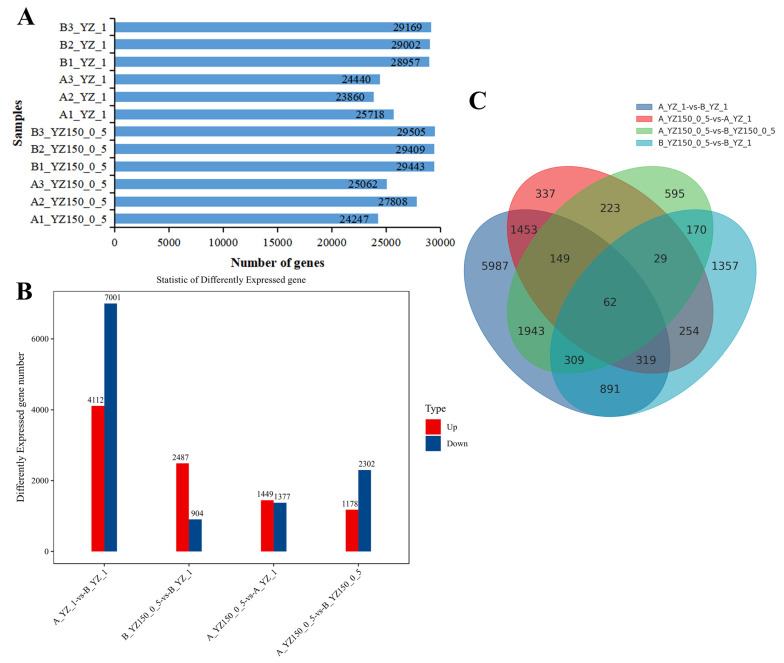
Total number of expressed genes and specific differentially expressed genes (DEGs) in different groups. (**A**) Total number of expressed genes in each sample; (**B**) Numbers of down- and up-regulated DEGs in different comparison samples. (**C**) Veen diagram for DEGs in different comparison samples.

**Figure 4:**
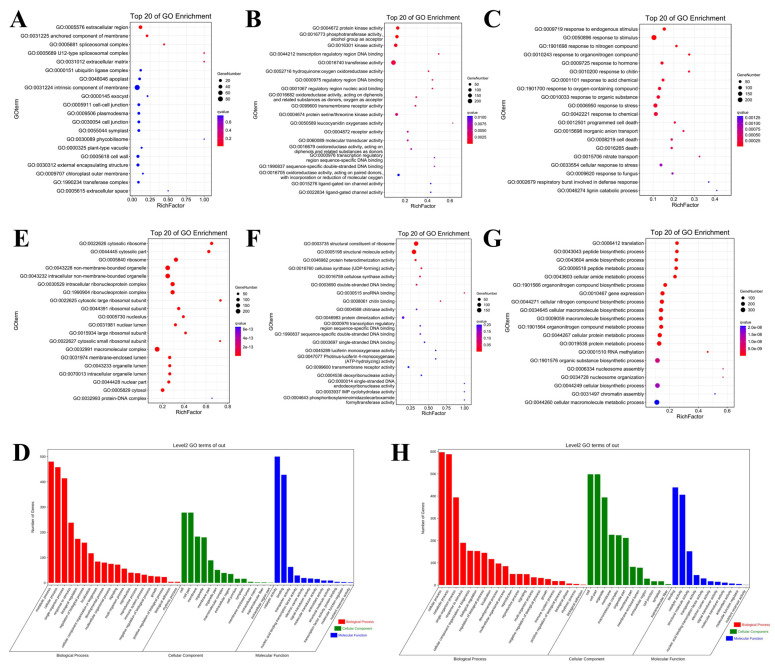
The Gene Ontology (GO) analysis and the top 20 of GO enrichment of DEGs in B-YZ150–0.5 vs. B-YZ−1 and A-YZ150–0.5 vs. A-YZ−1. The top 20 of GO enrichment of DEGs in (**A**) Cellular Component, (**B**) Molecular Function, (**C**) Biological Process and the (**D**) GO level secondary gene annotation in B-YZ150–0.5 vs. B-YZ−1; The top 20 of GO enrichment of DEGs in (**E**) Cellular Component, (**F**) Molecular Function, (**G**) Biological Process and the (**H**) GO level secondary gene annotation in B-YZ150–0.5 vs. B-YZ−1.

About 25,000 genes were detected from 12 libraries (Figure 3A). In order to investigate the dynamic changes between roots and protoplasts under the same treatment or in the same tissues under salt treatment and the control, different comparisons were performed by pairwise to identify the key DEGs, as: A_YZ_1-vs-B_YZ_1, B_YZ150_0.5-vs-B_YZ_1, A_YZ150_0.5-vs-A1_YZ_1, and A_YZ150_0.5-vs-B_YZ150_0.5. A total of 11,113 DEGs were identified in A_YZ_1-vs-B_YZ_1, with 4112 upregulated and 7001 downregulated genes; 3391 DEGs were found in B_YZ150_0.5-vs-B_YZ_1, with 2487 upregulated and 904 downregulated genes. In A_YZ150_0.5-vs-A1_YZ_1, the total number of DEGs were 2826 with 1449 upregulated and 1377 downregulated genes; there were 3480 DEGs in A_YZ150_0.5-vs-B_YZ150_0.5 with 1178 upregulated and 2302 downregulated genes (Figure 3B). Similar to the above-mentioned alignment rates, more DEGs were observed in the samples under normal growth conditions, and similar phenomenon also occurred before the protoplast dissociation, indicating that either salt stress or protoplast dissociation could decrease the number of DEGs. Additionally, Venn diagrams were plotted to obtain unique and common DEGs under salt-treated and between roots and protoplasts (Figure 4C); there were 1943 common DEGs between A_YZ_1-vs-B_YZ_1 and A_YZ150_0.5-vs-B_YZ150_0.5, and 254 common DEGs between B_YZ150_0.5-vs-B_YZ_1 and A_YZ150_0.5-vs-A1_YZ_1. A total of 62 DEGs were contained among the pairwise comparisons libraries of the four libraries, which were regarded as the core DEGs and subsequent functional enrichment analyses.

GO analysis was performed to cluster the DEGs, which were classified into three enriched categories: cellular component, biological process, and molecular function. In the cellular component, the identified DEGs were mainly mapped to cell, cell part, organelle, and membrane (Figure 4A,D,E,H). These DEGs were mainly enriched in cellular process, metabolic process, single-organism process, and response to stimulus in the biological process (Figure 4C,D,G,H). In B_YZ150_0.5-vs-B_YZ_1, most DEGs were mapped to catalytic activity, binding, transporter activity and nucleic acid binding transcription factor activity (Figure 4B,D), while those gathered in binding, catalytic, structural molecular activity, and transporter activity in A_YZ150_0.5-vs-A1_YZ_1 (Figure 4F,H). Due to the fact that many genes were associated with metabolic process, response to stimulus, electron carrier activity and signal transducer activity, these genes might play an important role in cotton response to salinity stress. 

KEGG pathway analysis was performed to screen the significant signal pathways during cotton response to salinity stress; the top 20 pathways were dominantly enriched in B_YZ150_0.5-vs-B_YZ_1 and A_YZ150_0.5-vs-A1_YZ_1 (Figure S2). In B_YZ150_0.5-vs-B_YZ_1, the plant hormone signal transduction, MAPK signaling pathway-plant, phenylpropanoid biosynthesis, biosynthesis of secondary metabolites, and alpha-Linolenic acid metabolism were the most enriched pathways (Figure S2A,B). The majority of DEGs were annotated into the ribosome, ribosome biogenesis in eukaryotes, phenylpropanoid biosynthesis, alanine, aspartate, and glutamate metabolism, and brassinosteroid biosynthesis in A_YZ150_0.5-vs-A1_YZ_1 (Figure S2C,D). KEGG pathway annotation was divided into metabolism, genetic information processing, environmental information processing, cellular processes, and organismal systems, and most DEGs were gathered in the same pathways between A_YZ150_0.5-vs-A1_YZ_1 and B_YZ150_0.5-vs-B_YZ_1, such as global and overview map and carbohydrate metabolism, carbohydrate metabolism and biosynthesis of other secondary metabolites in metabolism, signal transduction in environmental information processing, transport and catabolism in cellular processes, and environmental adaptation in organismal systems (Figure S2B,D). The only difference appeared in genetic information processing, and more DEGs were enriched in folding, sorting and degradation between A_YZ150_0.5-vs-A1_YZ_1, which yet gathered in translation from B_YZ150_0.5-vs-B_YZ_1 (Figure S2B,D). Combined with the above-mentioned KEGG pathways and previous results on plant response to salt stress, plant hormone signal transduction and MAPK signaling pathway were found in this study to contribute to salt-resistance in cotton roots, which should be subjected to further investigation for identifying the pivotal candidate genes and revealing the underlying genetic mechanisms. 

### 2.3. DEGs Involved in Plant Hormone Signal Transduction

**Figure 5:**
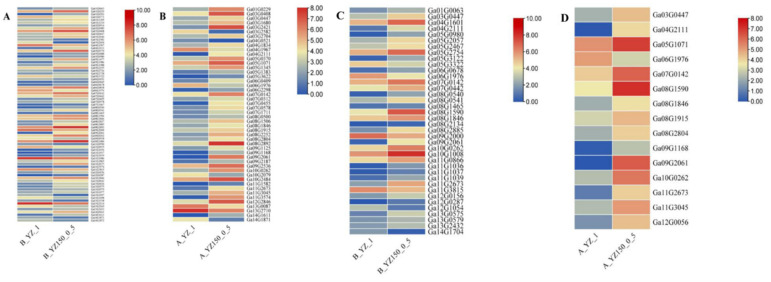
The expression patterns of genes involved in plant hormone signal transduction and MAPK signaling pathways. (**A**) B-YZ150–0.5 vs. B-YZ−1 and (**B**) A-YZ150–0.5 vs. A-YZ−1 show the gene expression involved in plant hormone signal transduction, and (**C**) B-YZ150–0.5 vs. B-YZ−1 and (**D**) A-YZ150–0.5 vs. A-YZ−1 show the gene expression involved in MAPK signaling pathways.

During the processes of plant growth and development, or responding to biotic and abiotic stresses, plant hormone signal transduction pathway plays important roles. Many plant hormone signal transduction pathway-related genes were found in this study to be differentially expressed under salt stress. There were 76 DEGs involved in plant hormone signal transduction in B_YZ150_0.5-vs-B_YZ_1 (Figure 5A and Table S1), including 54 upregulated and 22 downregulated DEGs, the auxin signaling pathway (17 genes), ABA signaling pathway (6 genes), Brassinosteroid signaling transduction pathway (2 genes), and ethylene (ETH) pathway (4 genes).

A total of 48 DEGs were involved in plant hormone signal transduction in A_YZ150_0.5-vs-A1_YZ_1, including 40 upregulated and 8 downregulated DEGs (Figure 5B and Table S2). These genes are involved in ABA signaling pathway (2 genes), auxin signaling pathway (13 genes), brassinosteroid signaling transduction pathway (2 genes), and ethylene (ETH) pathway (4 genes). Additionally, 18 common DEGs were observed in both fresh roots and dissociated protoplasts; their similar expression patterns showed that most DEGs firstly decreased in both roots and protoplasts before salt stress, then increased after salt stress treatment (Figure 5A,B). Interestingly, most DEGs belonging to auxin signaling pathway in protoplasts showed increased expression levels under salt stress, while the expressions were different in roots (Tables S1 and S2), indicating that auxin signaling pathway might be of great significance for protoplasts in response to salt stress. 

### 2.4. DEGs Involved in MAPK Signaling Pathway

MAPK signaling pathway was also found to be highly enriched in KEGG analysis; a total of 38 DEGs and 15 DEGs were identified in roots and protoplasts, respectively (Figure 5C,D), including the ABA signaling pathway (6 genes), Brassinosteroid signaling transduction pathway (2 genes) and ethylene (ETH) pathway (6 genes) (Tables S3 and S4). There were 32 upregulated and 6 downregulated DEGs in B_YZ150_0.5-vs-B_YZ_1, and 14 upregulated and 1 downregulated DEG in A_YZ150_0.5-vs-A1_YZ_1 associated with MAPK signaling pathways (Figure 5C,D).

As an endogenous hormone produced by plants in response to drought and salt stress, Abscisic acid (ABA) binds to the receptor protein PYR/PYL to relieve the inhibition of phosphatase PP2C on kinase SnRK2 activity and induces plant stress response by activating SnRK1 [30,31,32,33,34,35], which are also involved in MAPK signaling pathways and inhibits plant growth (Figures S1 and S2). In our study, seven genes were found to be involved in ABA signaling transduction in cotton response to salt stress, namely Ga06G1976 (*PYL6*), Ga04G2111 (Os01g0656200−1), Ga07G0142 (*ABI2*), Ga09G2061 (Os01g0656200−2), Ga10G0262 (*PP2CA*), Ga01G0063 (*SRK2E*), and Ga08G1915 (*SAPK3*) (Figure 5C,D). Among them, only PYL6 was downregulated while the other genes were upregulated in ABA signaling pathways to adapt to salt stress. 

### 2.5. Identification of Differentially Expressed TFs after Salt Treatment

**Figure 6:**
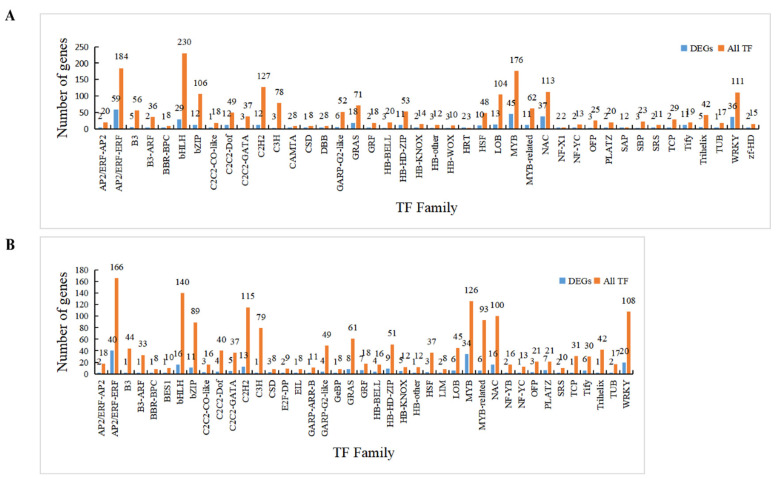
The distribution of TFs family with differential expression in (**A**) B-YZ150–0.5 vs. B-YZ−1 and (**B**) A-YZ150–0.5 vs. A-YZ−1. The *X*-axis represents the transcription factor (TF) families, and the *Y*-axis represents the number of genes.

TFs play an important role in plant response to abiotic stress [12,14]. In order to study their potential functions in cotton under salt stress, the obtained DEGs were subjected to the identification and difference analysis on TFs, resulting in 384 and 257 differentially expressed TFs in roots and protoplasts under salt stress, respectively (Figure 6 and Table S5). These TFs belong to 42 families, mainly including AP2/ERF-ERF, MYB, WRKY, NAC, bHLH, and GRAS, of which MYB, NAC, bHLH, and bZIP were reported as well-known TFs responsible for salt stress in plants [12]. Among the 384 differentially expressed TFs in roots, the majority belonged to the AP2/ERF-ERF family (59), followed by MYB (45), NAC (37), and WRKY (36), and the similar order of TF numbers were observed in protoplasts as: AP2/ERF-ERF (40), MYB (34), NAC (16), and WRKY (20). There were 293 up-regulated and 91 down-regulated TFs in roots, while 190 up-regulated and 67 down-regulated TFs were identified in protoplasts. These results showed that AP2/ERF-ERF had the largest proportion of TFs either in fresh roots or dissociated protoplasts, implying the potentially important functions in the cotton roots in response to salt stress. 

### 2.6. Validation of the RNA-Seq Results by qRT-PCR

**Figure 7:**
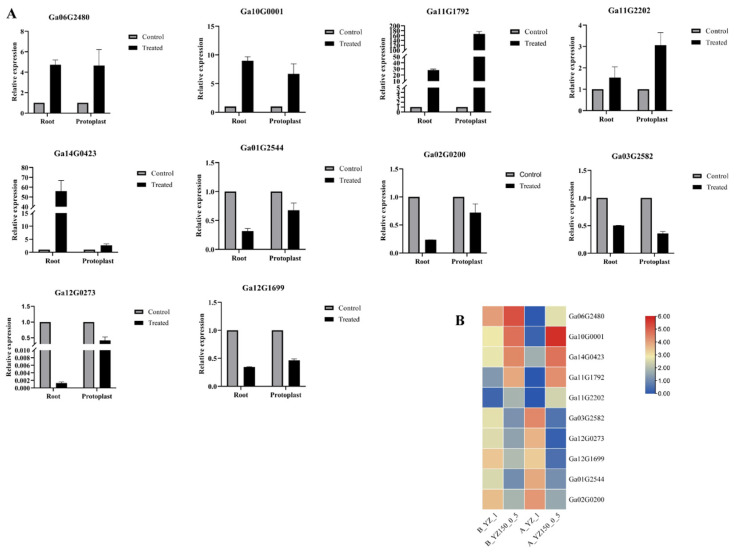
Quantitative real-time PCR (qRT-PCR) validation and RNA-seq data of ten selected DEGs. The figures show the (**A**) qRT-PCR data and the (**B**) RNA-seq data.

To verify the reliability of RNA-seq data, qRT-PCR was performed to confirm the transcript levels of ten randomly selected genes associated with salt stress. The gene-specific premiers were shown in Table S6, and three biological and three technical replicates for each gene were set up for subsequent analysis. The qRT-PCR results were consistent with the results of the RNA-seq data (Figure 7), directly proving the reproducibility of our transcriptome data.

## 3. Discussion

Cotton is an important cash crop and one of the main raw materials of textiles, which occupies an important position in the national economy and people’s life. Although being a pioneer salt tolerant crop, its limited salt tolerance seems insufficient to deal with the increasingly serious phenomenon of water shortage and salinization. Therefore, it is of great significance to cultivate cotton varieties with salt tolerance and drought resistance suitable for soil and environment in China. The root tip of cotton is the first part to respond to abiotic stress, so it is quite necessary to study the genes of root tip cells responding to abiotic stress for improving the salt tolerance and drought resistance of cotton. Till now, there are no reports in cotton on the transcriptome of roots protoplasts in response to salt stress.

As one of existing diploid cultispecies, *G. arboreum* harbors many merits of precocity, strong resistance, and high strength through long-term cultivation process, thus, not only contributing to 2–5% of world cotton yield every year, but aslo forming the famous Chinese cotton seed line. In consideration of clear genetic background, salt resistance, and complete genomic information [36,37,38], this important cotton genus was taken as our subject to perform protoplast dissociation and RNA-seq analysis. Moreover, Plants with medicinal potential and their secondary metabolites have been identified and widely applied in the earliest records of human habitation; herbology and advanced medicine in ancient systems have created one of the most important scientific foundations for safety in all areas of humanity [39]. Similarly, cotton also has certain medicinal value, such as the cotton wool on the seeds of *G. herbaceum*, *G. hirsutum*, and *G. barbadense*, which, as medicinal parts, have the effects of hemostasis and blood vomiting. These reports strengthen our determination to excavate more useful information on cotton plants responding to salt stress and presenting medicinal value, which needs further experiments for validation and application in the future.

The frequent occurrence of extreme weather has intensified the adverse effects of abiotic stresses on agricultural production [40]; as one of the most important abiotic environmental conditions, salt stress could seriously affect plant growth and production. Salinization limits not only the usable areas of planting cereals, but also the plant types of economic crops, synergistically causing yield and quality reductions. Additionally, salt stress causes osmotic stress and ion toxicity to cells, and its secondary effects will lead to oxidative stress and damage of cell components such as membrane, protein, and nucleic acid [41]. Compared with plant stems and leaves, root tips are the important tissues that are in direct contact and that respond first to salt stress. In this study, cotton root tips and its dissociated protoplasts were chosen to conduct transcriptome sequencing under normal growth condition and salt stress, aiming at revealing the molecular mechanisms of cotton plant root response to salt stress.

Due to the existence of cell walls, plant cells are not as easy as animal cells to perform many experiments on, such as molecular-biology experiments and scRNA-seq experiments, of which the former caused the limited transient genetic transformation and interaction protein identification, while the latter hindered the wide application of cellular heterogeneity research. The dissociated protoplasts rose in response to the proper time and conditions for resolving the above-mentioned bottlenecks; therefore, it appears quite important to obtain the high-quality protoplasts using a mild enzymolysis solution to dissociate the plant cell wall. Plenty of common RNA-seq have been performed on plant tissues to investigate the cellular types responding to growth and development or biotic and abiotic stresses. Little substantial progress might result from low inter-cell heterogeneity of plants, causing many marker genes obtained from bulk-seq to be unreliable and unable to be used for subsequent cell populations. The scRNA-seq, focusing on single cells, aims at detecting the dynamic variations of gene expressions during the specific biological processes. Sequencing on the high-quality protoplasts can solve the low heterogeneity between plant cells, promoting that the specific cell types could be more directly detected in response to the corresponding stresses, especially at the single cell level. Hence, it is of great significance to perform Bulk RNA-seq on the dissociated protoplasts in order to lay solid foundations for further scRNA-seq. 

Referring to the protoplast dissociating method of *Arabidopsis* root [17], we developed a highly-efficient method specific for cotton root protoplasts, which was subjected to four aspect evaluation, namely as root age, enzymatic hydrolysis time, vacuum treatment time, and salinity treatment time. By comparing the quantity and quality of the dissociated protoplasts from cotton roots on 5th, 7th, 9th, and 11th day, only 5-day-old samples obtained 2.0 × 10^6^ protoplasts with over 85% of cell viability; different time of enzymatic hydrolysis also gave different results, which showed that cotton roots began to dissociate protoplasts from 2 h, then gradually increased their amount and viability from 2 h to 6 h and reached the highest viability (more than 85%), and finally decreased at 8 h; 0.05 MPa vacuum treatment was applied to improve the dissociation efficiency and to confirm the whole cellular types. Current results showed that 1 h vacuum treatment leads to more high-efficiency than 0.5 h vacuum treatment and higher viability than 2 h vacuum treatment, which also met the requirement of scRNA-seq; based on the previous cotton treatment with 150 mM salinity [42,43] and the pre-experiments with different salinity, MS liquid medium was utilized to detect the viability under salt stress from 0 h to 2 h, and 2.0 × 10^6^ protoplasts were obtained with over 85% viability under 0.5 h treatment. Based on this study, the 5-day-old lateral root tips subjected to vacuum treatment for 1 h and digestion time for 6 h to obtain the high-quality protoplasts for RNA-seq.

Hormones regulate plant growth and development, of which abscisic acid signal transduction pathway is considered as the core regulating plant response to drought and salt stress [35]. Through inducing the expression of growth regulation genes, auxin could control the development of rice panicles and spikelets as well as the grain filling process [44]. In this study, numerous differentially expressed genes were found to be enriched in plant hormones signal transduction pathways, indicating that these might play important roles in the response to salt stress. There were 18 common DEGs identified in root tissue and protoplast, mainly including Ga06G1976 (abscisic acid receptor PYL4-like), Ga03G2582 (auxin-induced protein 15A), Ga14G1611 (auxin-responsive protein IAA20-like isoform X1), Ga07G0455 (auxin-responsive protein IAA29-like), Ga07G0578 (auxin-responsive protein SAUR40), Ga03G0447 (ethylene-responsive transcription factor 1B-like), Ga12G2846 (gibberellin receptor GID1B-like), Ga03G0408 (hypothetical protein GOBAR_AA09893), Ga11G3574 (probable indole-3-acetic acid-amido synthetase GH3.1), Ga10G0262 (protein phosphatase 2C 37-like), Ga10G2484 (protein TIFY 11B-like), Ga08G1506 (putative indole-3-acetic acid-amido synthetase GH3.5 ) and Ga08G1846 (transcription factor MYC2-like). The majority of these genes were significantly up-regulated after salt stress, indicating that they might be involved in salt stress and that they might play a certain regulatory role in cotton response to salt stress.

A large number of MAP kinase pathway family members exists in plants, which can combine each other to form thousands of different MAP kinase modules [36]. For example, *A. thaliana* contains more than 60 MAP trikinase (MAP3K), 10 MAP dikinase (MAP2K) and 20 MAP kinase (MAPK) [45]. For a long time, multiple MAPKs, mainly the rapid activation of MPK3, 4 and 6, have been observed in plants responding to abiotic and biotic stresses, such as salt, drought, and cold stress, and responding to growth and development signals. Salt, drought, and osmotic stresses rapidly activate SnRK2 family protein kinases, and all 10 SnRK2s except for SnRK2.9 could be activated by ABA in *Arabidopsis* [46]. In this study, upstream genes regulating SnRK2 were found as DEGs involved in MAPK signal transduction, including Ga06G1976 (*PYL6*), Ga04G2111 (Os01g0656200), Ga07G0142 (*ABI2*), Ga09G2061 (Os01g0656200), Ga10G0262 (*PP2CA*), Ga01G0063 (*SRK2E*) and Ga08G1915 (*SAPK3*), which were mainly involved in the regulation of SnRK2 kinase in plant growth and stress response. ABA binding to receptor protein PYL can relieve the inhibition of phosphatase PP2C on kinase SnRK2 activity, activate SnRK2, induce plant stress response, inhibit growth, and may be involved in the downstream MAPK signaling pathway, making cotton adapt to salt stress.

A method for cotton root tip protoplast dissociation developed in this study can not only be applied to cotton scRNA-seq, but also to study the cell specificity when facing different environmental stresses, including abiotic stress. In addition, the application of protoplasts in the genomics and epigenomics of cotton opens a new way to study the heterogeneity of cells in a variety of biological processes. Therefore, this study is of great significance in single cell sequencing.

## 4. Materials and Methods

### 4.1. Plant Materials and Growth Conditions

The *G. arboreum* L. accession Shixiya1 (SXY1) was used in this study, and were obtained from Chinese Academy of Agricultural Sciences (Anyang, Henan, China). Seeds were firstly sterilized with 0.1% HgCl_2_ for 15 min and risen by sterilized ddH_2_O for at least three times. The sterilized seeds were germinated on Murashige and Skoog (MS) medium with 30 g glucose and 5.2 g/L phytagel, pH 5.7. The seedlings were grown in a growth chamber at 28 °C (day)/25 °C (night) in long-day conditions (16 h light/8 h dark). On the 5th day, the seedlings were randomly divided into four groups, of which the two groups were treated with MS liquid medium containing 40 mL of 150 mM NaCl solution [42,43], and the other two groups were treated with 40 mL MS liquid medium only as control. The root samples were harvested from each treatment and were divided into two groups, one used for protoplast dissociation and another one for directly isolating RNAs. Each experiment was replicated three times.

### 4.2. Protoplast Isolation 

Due to the relatively smaller number of axial roots, the 0.5–1 cm region of lateral root tips was collected from the 5th days-old seedlings for isolating protoplasts. Selected lateral root sections (~1 g) were cut into pieces about 2 mm in diameter, and then all the sections were transferred into 10 mL sterilized enzyme solution (1.5% cellulase R10, 1% Pectolyase, 0.4 M mannitol, 0.1 M KCl, 0.08 M MES, 0.02 M CaCl2 and 0.1% BSA). After vacuum treatment under dark for 1 h, the samples and enzyme solution were carried out in the dark for 6 h with gentle shaking (80 r/min) at 25 ℃. The dissociated protoplasts were filtered with a cell strainer (40 μm diameter), concentrated, and washed two to four times with WB buffer (0.1 M KCl, 0.08M MES, 0.4 M mannitol and 0.1% BSA) at room temperature. 

### 4.3. Protoplast Counting and Viability

The high-quality protoplasts are the foundation of further genetic transformation and scRNA-seq, of which the quantity and quality were evaluated by hemocytometer counting and Trypan Blue staining, respectively. Not just the density of protoplasts, but also their cell viability and fragmentation rate, were calculated, and the counting procedure is as follows: ten microliters of protoplast solution was firstly added on the surface of the hemocytometer, and the cover glass was then carefully placed to avoid water bubbles; using the ordinary optics microscope, the protoplasts were finally counted by red blood cell counting method. The density of protoplasts was calculated as: 

Number of protoplasts mL^−1^ = (sum of 80 cells ÷ 80) × 400 × 10^4^ × dilution ratio. 

Meanwhile, protoplast suspension was mixed with Trypan blue solution in a ratio of 5:1 and dyed for 3 min, and a small number of stained cells were absorbed and counted with a hemocytometer. Dead cells presented blue, swollen, and dull, while living cells could not be stained and remained normal and shiny. With the help of COSSIM FR-2L fluorescence microscope, the detected images were collected, and the number of active protoplasts and total protoplasts in each field were counted and the viable rate of protoplasts (%) was calculated as follows: 

Viable protoplast (%) = (number of living protoplasts)/(number of living cells + number of dead protoplasts) × 100%. 

### 4.4. RNA Isolation, Library Construction, and Sequencing

Cotton seedlings at 5th day were treated with 150 mM NaCl for half an hour, and protoplasts obtained from lateral root tips and root tips before and after treatment were collected for RNA extraction. More than 1 × 10^6^ protoplasts were collected, centrifuged, only protoplast precipitation was retained for RNA isolation. About 0.1 g fresh roots were also sampled for RNA isolation. Total RNA was extracted with an RNAprep Pure Plant Kit (Tiangen, Beijing, China) from root samples or the protoplasts, following the instructions of the manufacturer. The quality and the concentration of each RNA sample were determined by NanoDrop 2000 spectrophotometer and RNA that fulfilled the standard at 260/280 in a range of 1.80–2.1 was used for further analysis [47]. The RNA quality was also determined by agarose gel electrophoresis and spectrometric analysis. Subsequently, the high-quality RNA was used for library construction and transcriptome sequencing commercially performed by Shanghai OE Biotech (Shanghai, China). Three biological replicates were performed for each group with a total of 12 cDNA libraries. Finally, Illumina HiSeq 2500 (Illumina, San Diego, CA, USA) sequencing platform was utilized for RNA-seq on each cDNA library.

### 4.5. Data Quality Control and Identification and Enrichment Analyses of DGEs

All RNA-seq data were deposited in the National Center for Biotechnology Information (NCBI). Trimmomatic (version 0.36) [48] was used to process the raw data. After removing low quality sequences, the obtained clean reads were mapped to the *G. arboreum* reference genome [37] using HISAT2 version 2.2.1.0 [49]. 

The fragments per kb per million reads (FPKM) was used to represent the gene expression, and differentially expressed genes (DEGs) were identified by the cufflinks (version 2.2.1) [50]. DESeq (version 1.18.0) was used to standardize the counts number of each sample gene (BaseMean value was used to estimate the expression levels), the multiple of difference was calculated, and NB (negative binomial distribution test) was used to test the significance of difference. Finally, the differential protein coding genes were screened according to the difference multiple and difference significance test results. Additionally, enrichment analyses were performed on the identified DEGs in order to investigate the key functional genes or signal pathways regulating plant root response to salt stress, which were annotated by Gene Ontology analysis (GO, http://geneontology.org/, accessed on 24 December 2021) [51] and Kyoto Encyclopedia of Genes and Genomes analysis (KEGG, https://www.genome.jp/kegg/, accessed on 7 December 2021) [52].

### 4.6. Quantitative Real-Time PCR (qRT-PCR) Verification

The total RNA was extracted from fresh plant root tips and their dissociated protoplasts with an RNAprep Pure Plant Kit (Tiangen, Beijing, China), which subsequently underwent reverse transcription using TransScript-All-in-One First-Strand cDNA Synthesis SuperMix (TransGen Biotech kit, Beijing, China) and TransStart Top Green qPCR SuperMix (TransGen Biotech) for RT-qPCR in harmony with the protocol of the manufacturer. The cDNA was diluted 10-fold and used as templates for qRT-PCR, which was performed in an ABI 7500 qRT-PCR system (Applied Biosystems, Foster City, CA, USA). Total reaction mixture volume was 20 μL containing 2 μL cDNA, 6.6 μL Nuclease-free water, 10 μL Green qPCR SuperMix, 0.4 μL Dye, 0.5 μL of each forward and reverse primer. The cotton *Ubiquitin7* gene (*(UB7*, GenBank: DQ116441) was used as a reference gene [53], and the relative expression changes of targeted genes were evaluated by the 2^△△^^Ct^ method [54]. qRT-PCR experiments were conducted with three biological repeats and three technological repeats on 10 randomly selected genes.

## 5. Conclusions

For the first time, we report the highly-efficient method for protoplast dissociation from lateral roots of cotton under salt stress. Using this method, a high quality and quantity of protoplasts were obtained for scRNA-seq. Meanwhile, RNA-seq was conducted on the cotton root tips of 5-day-old seedlings and its dissociated protoplasts under salt and control conditions, and 22 and 9 common DEGs responsible for salt stress were found to participate in plant hormone signaling and MAPK signaling between the samples before and after dissociation. There were six upregulated DEGs involved in ABA signaling transduction, indicating their potential functions on plant adaption to salt stress, namely as Ga04G2111, Ga07G0142, Ga09G2061, Ga10G0262, Ga01G0063, and Ga08G1915. Additionally, 384 and 257 transcription factors were differentially expressed in salt-stress roots before and after protoplast dissociation, respectively, of which significantly up-regulated TFs mainly belong to the AP2/ERF-ERF family. These results not only provide important information for the exploration of single cell transcriptome sequencing in cotton and the molecular mechanisms of cotton in response to salt stress, but also lay a solid foundation for breeding novel varieties harboring high-yield, superior-fiber, and multiple-resistance traits. 

## Figures and Tables

**Table 1:** RNA-seq analysis of cotton under salt-stress.

Sample	Raw Reads (M)	Clean Reads (M)	Q30 (%)	GC Content (%)	Total Mapped (M) (%)
A1_YZ_1	48.55	47.59	93.88	45.79	41.78 (87.79)
A2_YZ_1	50.18	49.24	94.04	44.82	46.66 (94.77)
A3_YZ_1	51.72	50.68	93.99	44.85	48.33 (95.36)
B1_YZ_1	49.31	47.95	91.85	44.36	47.14 (98.32)
B2_YZ_1	48.73	47.48	91.96	44.48	46.65 (98.27)
B3_YZ_1	48.95	47.61	91.85	44.29	46.84 (98.37)
A1_YZ150_0_5	46.63	44.18	92.46	45.86	38.96 (88.20)
A2_YZ150_0_5	51.23	50.22	93.36	45.56	45.27 (90.15)
A3_YZ150_0_5	47.36	44.63	92.19	46.38	38.51 (86.28)
B1_YZ150_0_5	51.00	49.97	93.37	44.55	48.97 (97.99)
B2_YZ150_0_5	48.32	47.38	93.46	44.51	46.54 (98.23)
B3_YZ150_0_5	47.17	46.31	93.63	44.43	45.43 (98.10)

Notes: A_YZ_1 and B_YZ_1 represent the protoplast of lateral root tips and the lateral root tips without salt treatment, respectively. A_YZ150_0_5. B_YZ150_0_5 represent the protoplast of lateral root tips and the lateral root tips with salt treatment, respectively. A1, A2, A3, B1, B2 and B3 stand for the three biological replicates. M represents million. Q30 stands for the percentage of nucleotides with a quality value ≥ 30. GC Content represents the percentage of guanine and cytosine in the clean reads.

## Data Availability

Not applicable.

## Supplementary Materials

The following supporting information can be downloaded at: https://www.mdpi.com/article/10.3390/ijms23052845/s1.
