# Supplementary material for: Protoplast Dissociation and Transcriptome Analysis Provides Insights to Salt Stress Response in Cotton
Source: Int J Mol Sci. 2022 Mar 5;23(5):2845. doi: 10.3390/ijms23052845 (PMC8911145; doi:10.3390/ijms23052845)
Supplement: Supplementary file 1 [file ijms-23-02845-s001.zip › ijms-1572004-supplementary.pdf]

Table S1. The expression of DEGs (FPKM) involved in hormone signal transduction pathways in B-YZ150-0.5 vs B-YZ-1.

Gene_ID	B-YZ-1	B-YZ150-0.5	Description
Ga05G3322	25.57371881	9.716549987	abscisic acid receptor PYL4-like
Ga06G1976	53.83743675	14.70281589	abscisic acid receptor PYL4-like
Ga09G2000	108.7117546	41.48460459	abscisic acid receptor PYL4-like
Ga11G3815	66.78363899	33.77376668	abscisic acid receptor PYR1-like
Ga14G0161	14.2353155	38.36601993	abscisic acid-insensitive 5-like protein 4
Ga05G2155	22.70095647	59.82489037	ABSCISIC ACID-INSENSITIVE 5-like protein 5
Ga01G1210	8.173722958	16.76218109	auxin response factor 5-like isoform X2
Ga03G2582	5.479198635	1.222000419	auxin-induced protein 15A
Ga05G2726	5.319301088	1.855757744	auxin-induced protein 15A-like
Ga13G2114	286.6109818	96.43372682	auxin-induced protein 22B-like
Ga11G1086	252.0634174	97.22643683	auxin-induced protein 22D-like
Ga01G1209	5.752395076	20.08282563	auxin-induced protein 6B-like
Ga05G1988	3.46285887	20.144702	auxin-induced protein 6B-like
Ga05G2728	2.692182645	6.35828607	auxin-induced protein 6B-like
Ga13G1077	3.823815065	8.670472195	auxin-induced protein 6B-like
Ga08G2827	2.227000813	8.347517115	Auxin-responsive IAA16 -like protein
Ga14G1611	0.909668536	7.379160435	auxin-responsive protein IAA20-like isoform X1
Ga07G0455	1.711294806	8.063905021	auxin-responsive protein IAA29-like
Ga05G0488	1.559192158	3.256060066	auxin-responsive protein IAA6-like
Ga13G1097	5.237786604	0.400534769	auxin-responsive protein SAUR23-like
Ga13G1098	18.10298989	6.373468301	auxin-responsive protein SAUR23-like
Ga07G0578	4.171007644	10.75452944	auxin-responsive protein SAUR40
Ga14G1872	6.323443594	1.92739558	auxin-responsive protein SAUR72-like
Ga13G0579	2.326574605	5.236785468	BRASSINOSTEROID INSENSITIVE 1-associated receptor kinase 1-like
Ga13G0575	1.460967382	8.875397204	BRASSINOSTEROID INSENSITIVE 1-associated receptor kinase 1-like isoform X1
Ga07G0442	14.1799664	40.5368136	EIN3-binding F-box protein 1-like
Ga03G0447	6.559928856	24.80320072	ethylene-responsive transcription factor 1B-like
Ga11G1037	0.127924426	1.216932521	ethylene-responsive transcription factor 1B-like
Ga11G1039	0.582010574	3.678440583	ethylene-responsive transcription factor 1B-like
Ga11G2673	3.129061137	45.77286782	ethylene-responsive transcription factor 1B-like
Ga12G2846	32.33397595	66.53658505	gibberellin receptor GID1B-like
Ga11G0935	40.03841371	14.41277103	gibberellin receptor GID1C-like

Ga13G2169	18.86786539	7.023892009	gibberellin receptor GID1C-like
Ga02G0914	2.880605506	6.773327207	histidine kinase 3-like
Ga08G0541	5.618528107	21.04439736	hypothetical protein B456_004G057100
Ga13G0042	6.753550563	17.53769702	hypothetical protein F383_22932
Ga13G1538	3.032886593	0.902947473	hypothetical protein GOBAR_AA00105
Ga03G2604	12.72810278	0.228984943	hypothetical protein GOBAR_AA04603
Ga03G0408	51.86866869	168.0951199	hypothetical protein GOBAR_AA09893
Ga05G1986	1.043787467	6.832476141	hypothetical protein GOBAR_AA12303
Ga08G0540	0.252282554	3.356802477	hypothetical protein GOBAR_AA23749
Ga01G0063	2.159505592	5.224609707	hypothetical protein GOBAR_AA30878
Ga11G1036	0.152130725	2.501173042	hypothetical protein GOBAR_AA35152
Ga06G0191	5.855253177	2.832967938	hypothetical protein GOBAR_AA38614
Ga14G1871	4.162804236	0.921461676	hypothetical protein GOBAR_DD00296
Ga11G0590	2.612169098	1.312980166	indole-3-acetic acid-amido synthetase GH3.17-like isoform X1
Ga01G0771	35.66580794	15.69854883	indole-3-acetic acid-amido synthetase GH3.6
Ga08G2892	63.23242192	263.8648874	JAZ4, partial
Ga03G2040	5.389792852	28.57753897	JAZ8, partial
Ga11G3574	0.401260164	1.008803993	probable indole-3-acetic acid-amido synthetase GH3.1
Ga13G0436	1.80713595	8.939137362	probable indole-3-acetic acid-amido synthetase GH3.1
Ga05G0980	0.146056243	3.254513037	probable protein phosphatase 2C 24
Ga04G2111	0.899425402	11.34766543	probable protein phosphatase 2C 51
Ga09G2061	0.308903834	17.22603326	probable protein phosphatase 2C 51
Ga12G0287	0.031217386	0.974121785	probable protein phosphatase 2C 8 isoform X1
Ga10G0262	21.58417622	82.96984927	protein phosphatase 2C 37-like
Ga06G0678	0.645500471	3.184808462	protein phosphatase 2C 56-like
Ga13G2432	2.771613767	6.340402512	protein phosphatase 2C 56-like
Ga08G2885	2.073792023	20.10591303	protein phosphatase 2C 77 isoform X1
Ga07G0142	39.07855425	90.80293248	Protein phosphatase 2C 77 -like protein
Ga05G0370	25.67464839	127.0726337	Protein TIFY 10A -like protein
Ga06G0838	36.86232083	89.33816111	Protein TIFY 10A -like protein
Ga05G1477	6.894417099	44.37328635	protein TIFY 11B-like
Ga10G2484	62.86850952	275.3061493	protein TIFY 11B-like
Ga02G0989	11.99416917	43.61655102	Protein TIFY 6B -like protein
Ga01G0229	37.83648341	114.8232399	protein TIFY 9
Ga08G1506	29.54229061	65.53856121	putative indole-3-acetic acid-amido synthetase GH3.5
Ga12G0156	2.594168555	8.316758609	putative phosphatase 2C 75 -like protein
Ga08G1465	0.7303442	1.553998262	transcription factor MYC2-like
Ga08G1846	26.83925248	56.0032048	transcription factor MYC2-like

Ga07G1467	0.478405844	1.089155799	transcription factor PIL1-like
Ga10G0918	15.37183088	6.913112271	two-component response regulator ARR17
Ga01G0301	0.143823073	4.028018887	Two-component response regulator ARR2 -like protein
Ga04G1967	92.94036542	34.51041877	two-component response regulator ARR8-like
Ga06G0445	0.521606584	1.430815138	two-component response regulator ORR9-like
Ga11G1865	7.525045998	19.90395736	Uncharacterized protein F383_10696

Table S2. The expression of DEGs (FPKM) involved in hormone signal transduction pathways in A-YZ150-0.5 vs A-YZ-1.

Gene_ID	A-YZ-1	A-YZ150-0.5	Description
Ga06G1976	32.0849278	7.148303846	abscisic acid receptor PYL4-like
Ga11G3045	4.487152858	33.87561061	abscisic acid receptor PYL9-like
Ga06G0409	1.508315296	11.54122581	auxin transporter-like protein 3
Ga03G2582	19.81843306	0.460543558	auxin-induced protein 15A
Ga09G2536	29.21172993	95.73814868	auxin-induced protein 22D-like
Ga05G1345	8.489909637	47.26299063	auxin-induced protein AUX22-like
Ga06G2298	0.173683233	2.976303127	auxin-induced protein AUX22-like
Ga05G1383	0.999339594	5.011463228	Auxin-responsive IAA27 -like protein
Ga07G1711	1.649394261	8.774124348	auxin-responsive protein IAA11
Ga08G2252	2.539958167	35.53806951	auxin-responsive protein IAA11-like isoform X1
Ga14G1611	0	4.200040969	auxin-responsive protein IAA20-like isoform X1
Ga07G0455	0.130099914	16.16992772	auxin-responsive protein IAA29-like
Ga05G3622	0	1.62772008	auxin-responsive protein IAA32-like isoform X2
Ga09G2187	0.739494829	5.604915782	auxin-responsive protein SAUR36-like
Ga07G0578	1.954992687	18.40634963	auxin-responsive protein SAUR40
Ga10G2079	16.06527904	3.647182401	cyclin-D3-3-like isoform X1
Ga08G2804	3.565260459	16.39365045	EIN3-binding F-box protein 1-like isoform X1
Ga03G0447	3.726235532	20.84863179	ethylene-responsive transcription factor 1B-like
Ga11G2673	0.891420582	16.5580022	ethylene-responsive transcription factor 1B-like
Ga03G2704	5.291331032	22.04703921	G-box-binding factor 4-like
Ga12G2846	11.1682482	127.060473	gibberellin receptor GID1B-like
Ga03G1680	5.711896004	30.04944182	Histidine kinase 3 -like protein
Ga13G2710	240.5376747	68.81351707	hypothetical protein GOBAR_AA06134
Ga03G0408	15.89866853	129.7393006	hypothetical protein GOBAR_AA09893
Ga14G1871	13.65854285	1.048500379	hypothetical protein GOBAR_DD00296
Ga04G1834	4.808233722	18.65009571	indole-3-acetic acid-amido synthetase GH3.17-like
Ga08G2892	10.83360274	213.979599	JAZ4, partial
Ga09G1168	0.099531909	5.143417533	pathogenesis-related protein 1-like
Ga03G2421	2.097233041	149.7959517	probable indole-3-acetic acid-amido synthetase GH3.1
Ga11G3574	0.113482788	52.72009796	probable indole-3-acetic acid-amido synthetase GH3.1
Ga04G2111	0.014604842	14.35808369	probable protein phosphatase 2C 51
Ga09G2061	0	79.43846533	probable protein phosphatase 2C 51

Ga05G1071	37.90288949	115.9121314	protein ETHYLENE INSENSITIVE 3
Ga10G0262	4.68145746	53.73696313	protein phosphatase 2C 37-like
Ga07G0142	16.97802322	71.18168524	Protein phosphatase 2C 77 -like protein
Ga05G0370	7.020978914	55.39866476	Protein TIFY 10A -like protein
Ga10G2484	39.04210483	139.299683	protein TIFY 11B-like
Ga01G0229	4.662374643	62.36018527	protein TIFY 9
Ga08G0500	1.578560014	5.896528275	protein TRANSPORT INHIBITOR RESPONSE 1-like
Ga08G1506	5.528043096	26.82749451	putative indole-3-acetic acid-amido synthetase GH3.5
Ga08G1915	7.236022336	22.4258122	serine/threonine-protein kinase SAPK3-like isoform X2
Ga04G0521	1.857150137	0.036747842	transcription factor APG-like
Ga08G1846	3.374685442	12.94582425	transcription factor MYC2-like
Ga11G1582	0	1.196082933	transcription factor TGA1-like isoform X2
Ga09G1125	2.408489391	11.72147366	transcription factor TGA2-like isoform X1
Ga07G0312	2.888025789	10.33673675	two-component response regulator ARR12-like
Ga04G1967	57.08486598	10.82870904	two-component response regulator ARR8-like
Ga13G0087	80.19486873	17.54172014	two-component response regulator ORR10

Table S3. The expression of DEGs (FPKM) involved in MAPK signaling pathways in B-YZ150-0.5 vs B-YZ-1.

Gene_ID	B-YZ-1	B-YZ150-0.5	Description
Ga05G3322	25.57371881	9.716549987	abscisic acid receptor PYL4-like
Ga06G1976	53.83743675	14.70281589	abscisic acid receptor PYL4-like
Ga09G2000	108.7117546	41.48460459	abscisic acid receptor PYL4-like
Ga11G3815	66.78363899	33.77376668	abscisic acid receptor PYR1-like
Ga13G0579	2.326574605	5.236785468	BRASSINOSTEROID INSENSITIVE 1-associated receptor kinase 1-like
Ga13G0575	1.460967382	8.875397204	BRASSINOSTEROID INSENSITIVE 1-associated receptor kinase 1-like isoform X1
Ga10G1008	60.04367576	273.6856357	calmodulin-like
Ga07G0442	14.1799664	40.5368136	EIN3-binding F-box protein 1-like
Ga03G0447	6.559928856	24.80320072	ethylene-responsive transcription factor 1B-like
Ga11G1037	0.127924426	1.216932521	ethylene-responsive transcription factor 1B-like
Ga11G1039	0.582010574	3.678440583	ethylene-responsive transcription factor 1B-like
Ga11G2673	3.129061137	45.77286782	ethylene-responsive transcription factor 1B-like
Ga08G0541	5.618528107	21.04439736	hypothetical protein B456_004G057100
Ga08G2134	0.118132569	0.397990333	hypothetical protein GOBAR_AA20802
Ga08G0540	0.252282554	3.356802477	hypothetical protein GOBAR_AA23749
Ga14G1704	2.586927503	0.214074975	hypothetical protein GOBAR_AA29146
Ga01G0063	2.159505592	5.224609707	hypothetical protein GOBAR_AA30878
Ga04G1601	35.71564796	116.8920483	hypothetical protein GOBAR_AA31266
Ga11G1036	0.152130725	2.501173042	hypothetical protein GOBAR_AA35152
Ga05G2467	7.606610451	19.04717426	mitogen-activated protein kinase kinase kinase 1-like
Ga12G1054	6.501517918	1.032079805	probable calcium-binding protein CML30
Ga05G3122	0.282246268	4.199215646	probable LRR receptor-like serine/threonine-protein kinase At3g47570
Ga05G0980	0.146056243	3.254513037	probable protein phosphatase 2C 24
Ga04G2111	0.899425402	11.34766543	probable protein phosphatase 2C 51
Ga09G2061	0.308903834	17.22603326	probable protein phosphatase 2C 51
Ga12G0287	0.031217386	0.974121785	probable protein phosphatase 2C 8 isoform X1
Ga10G0262	21.58417622	82.96984927	protein phosphatase 2C 37-like
Ga06G0678	0.645500471	3.184808462	protein phosphatase 2C 56-like
Ga13G2432	2.771613767	6.340402512	protein phosphatase 2C 56-like

Ga08G2885	2.073792023	20.10591303	protein phosphatase 2C 77 isoform X1
Ga07G0142	39.07855425	90.80293248	Protein phosphatase 2C 77 -like protein
Ga12G0156	2.594168555	8.316758609	putative phosphatase 2C 75 -like protein
Ga11G0866	20.8974268	48.60417264	respiratory burst oxidase homolog protein B
Ga05G2057	5.167486322	21.84856216	respiratory burst oxidase homolog protein D-like
Ga05G2754	39.00416779	111.4533515	respiratory burst oxidase homolog protein D-like
Ga08G1465	0.7303442	1.553998262	transcription factor MYC2-like
Ga08G1846	26.83925248	56.0032048	transcription factor MYC2-like
Ga08G1590	14.50186767	147.2874472	WRKY transcription factor 22-like

Table S4. The expression of DEGs (FPKM) involved in MAPK signaling pathways in A-YZ150-0.5 vs A-YZ-1.

Gene_ID	A-YZ-1	A-YZ150-0.5	Description
Ga06G1976	32.0849278	7.148303846	abscisic acid receptor PYL4-like
Ga11G3045	4.487152858	33.87561061	abscisic acid receptor PYL9-like
Ga08G2804	3.565260459	16.39365045	EIN3-binding F-box protein 1-like isoform X1
Ga03G0447	3.726235532	20.84863179	ethylene-responsive transcription factor 1B-like
Ga11G2673	0.891420582	16.5580022	ethylene-responsive transcription factor 1B-like
Ga12G0056	1.703225281	20.53358448	hypothetical protein GOBAR_AA23896
Ga09G1168	0.099531909	5.143417533	pathogenesis-related protein 1-like
Ga04G2111	0.014604842	14.35808369	probable protein phosphatase 2C 51
Ga09G2061	0	79.43846533	probable protein phosphatase 2C 51
Ga05G1071	37.90288949	115.9121314	protein ETHYLENE INSENSITIVE 3
Ga10G0262	4.68145746	53.73696313	protein phosphatase 2C 37-like
Ga07G0142	16.97802322	71.18168524	Protein phosphatase 2C 77 -like protein
Ga08G1915	7.236022336	22.4258122	serine/threonine-protein kinase SAPK3-like isoform X2
Ga08G1846	3.374685442	12.94582425	transcription factor MYC2-like
Ga08G1590	11.27363926	145.1899406	WRKY transcription factor 22-like

Table S5. Classification and number of up-regulated and down-regulated TFs in B-YZ150-0.5 vs B-YZ-1 and A-YZ150-0.5 vs A-YZ-1.

Name	B-YZ150-0.5 vs B-YZ-1		A-YZ150-0.5 vs A-YZ-1	
	Up	Down	Up	Down
AP2/ERF-AP2	2	0	1	1
AP2/ERF-ERF	48	11	34	6
B3	3	2	0	1
B3-ARF	2	0	1	0
BBR-BPC	1	0	0	1
BES1	0	0	1	0
bHLH	22	7	9	7
bZIP	6	6	10	1
C2C2-CO-like	1	0	3	0
C2C2-Dof	9	3	4	0
C2C2-GATA	3	0	0	5
C2H2	5	7	8	5
C3H	3	0	0	1
CAMTA	2	0	0	0
CSD	0	1	0	3
DBB	0	2	0	0
E2F-DP	0	0	0	2
EIL	0	0	1	0
GARP-ARR-B	0	0	1	0
GARP-G2-like	4	2	2	2
GeBP	0	0	0	1
GRAS	15	3	7	1
GRF	2	0	0	7
HB-BELL	1	2	4	0
HB-HD-ZIP	6	5	6	3
HB-KNOX	2	0	5	0
HB-other	3	0	1	0
HB-WOX	2	1	0	0
HRT	2	0	0	0
HSF	10	0	0	3
LIM	0	0	2	0
LOB	6	7	5	1
MYB	33	12	31	3
MYB-related	5	6	5	1
NAC	31	6	16	0
NF-X1	2	0	0	0
NF-YB	0	0	0	2
NF-YC	2	0	0	1
OFP	0	3	1	2
PLATZ	1	1	3	4

SAP	1	0	0	0
SBP	1	2	0	0
SRS	2	0	1	1
TCP	0	2	0	1
Tify	11	0	6	0
Trihelix	5	0	0	1
TUB	1	0	2	0
WRKY	36	0	20	0
zf-HD	2	0	0	0

Table S6. Primers used for qRT-PCR in this study.

Primer Names	Primer Sequences (5′–3′)
Ub7-F	5′- CTCCGAGAACGTCATCACCG-3′
Ub7-R	5′- TGGAGCCGTACTGGAACTGG-3′
Ga06G2480-F	5′- CAAAGTCAGCAGCACAGTGG-3′
Ga06G2480-R	5′- CTGCATCGACGAGGACTTGTA-3′
Ga10G0001-F	5′- CGCCGAGCAATTGAACGAAA-3′
Ga10G0001-R	5′- ACACCGTGGATCCTTTTCCC-3′
Ga14G0423-F	5′- CCATGCCCTGCTTGGACTAC-3′
Ga14G0423-R	5′- TCACAGTGCTACCAGCAAGT-3′
Ga11G1792-F	5′- CGCTTCGGGGTTCGATCATA-3′
Ga11G1792-R	5′- GACAAGGTTCCGGGACTGAG-3′
Ga11G2202-F	5′- TCAGTCAGCTCTCCGAAGGA-3′
Ga11G2202-R	5′- ATACTGCTCCTCGTCCTGGT-3′
Ga03G2582-F	5′- TCTCACCCCTCCCTTAGACG-3′
Ga03G2582-R	5′- GGAACTCGGGGTGAGTCAAG-3′
Ga12G0273-F	5′- CCGAATGTCAAGGCGAAACC-3′
Ga12G0273-R	5′- CCGACACCGAGGTGTTGAAA-3′
Ga12G1699-F	5′- AATGGAATGTCGGCAGTGGG-3′
Ga12G1699-R	5′- CAGGAGCTTCATGGGCAACT-3′
Ga01G2544-F	5′- ATGGGACGAGGACTCATGGA-3′
Ga01G2544-R	5′- ACCAATCACAGTCCGACACC-3′
Ga02G0200-F	5′- TTGCTTTGCCGTTGACTTGG-3′
Ga02G0200-R	5′- TTTCCGGCTTGGAGACTCAC-3′

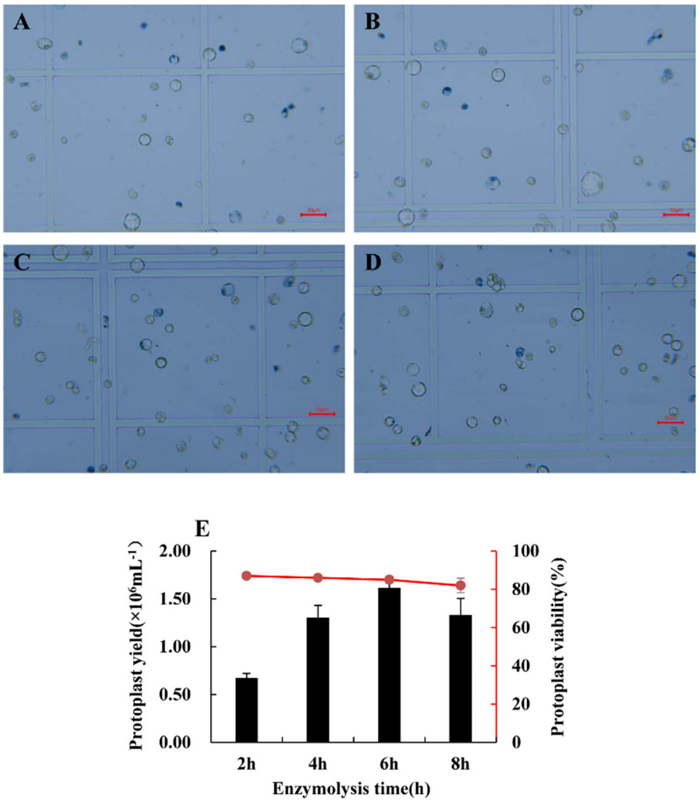

Figure S1 Effects of enzymolysis time on protoplasts isolation from *G. arboreum* lateral root tips. The protoplasts stained with Trypan blue solution, which were isolated from the enzyme digestion for **(A)** 2 h, **(B)** 4 h, **(C)** 6 h and **(D)** 8 h. Scale bar, 50 μm; **(E)** Effects of enzymolysis time on the yield and viability of protoplasts isolated from the cotton lateral root tips; data presented as means of three biological replicates with error bars indicating standard deviations (SD). The bar graph represents protoplast yield ($\times 10^6$) and the red broken line graph represents protoplast viability (%).

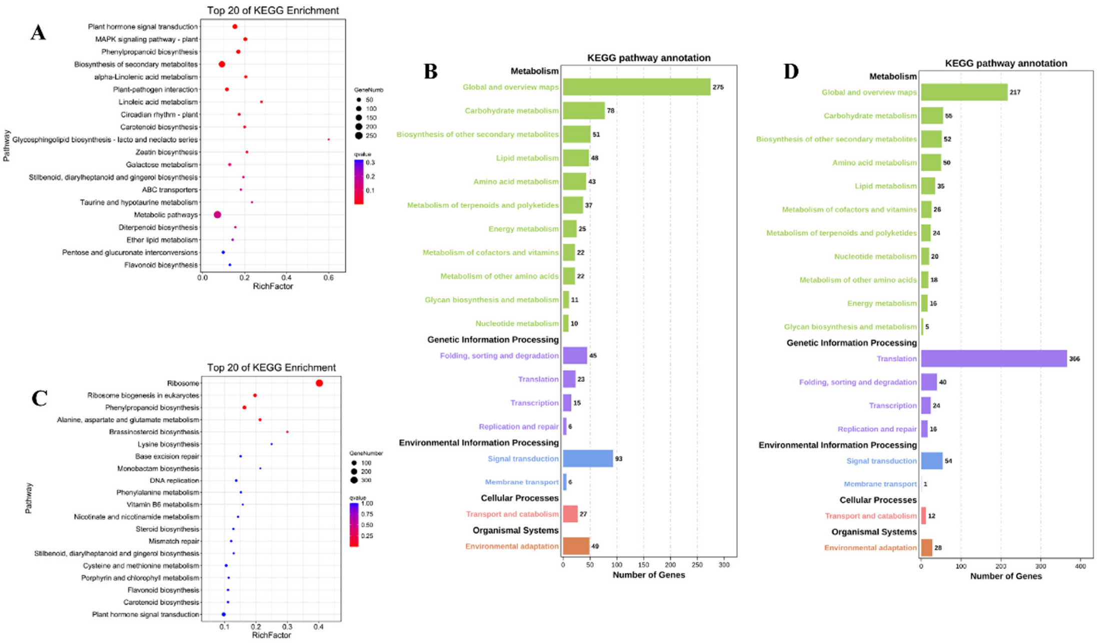

Figure S2 The Kyoto Encyclopedia of Genes and Genomes (KEGG) pathways analysis and the top 20 enriched pathways of DEGs in **(A)** B-YZ150-0.5 vs B-YZ-1 and **(B)** A-YZ150-0.5 vs A-YZ-1 and their pathways in **(C)** B-YZ150-0.5 vs B-YZ-1 and **(D)** A-YZ150-0.5 vs A-YZ-1.
